# Correlates of patient-reported cognitive performance with regard to disability

**DOI:** 10.1038/s41598-022-17649-3

**Published:** 2022-08-05

**Authors:** Delphine Van Laethem, Alexander De Cock, Jeroen Van Schependom, Ralph H. B. Benedict, Guy Nagels, Marie D’hooghe

**Affiliations:** 1 AIMS Lab, Center for Neurosciences, UZ Brussel, Vrije Universiteit Brussel, Pleinlaan 2, 1050 Brussel, Belgium; 2 Department of Electronics and Informatics (ETRO), Vrije Universiteit Brussel, Brussel, Belgium; 3 Department of Radiology, UZ Brussel, Brussel, Belgium; 4 University at Buffalo, Buffalo, NY USA; 5 Neurology Department, UZ Brussel, Brussel, Belgium; 6 Neurology Department, National Multiple Sclerosis Center, Melsbroek, Belgium; 7 St Edmund Hall, University of Oxford, Oxford, UK; 8 Center for Neurosciences, Vrije Universiteit Brussel, Brussel, Belgium

**Keywords:** Cognitive neuroscience, Multiple sclerosis

## Abstract

The patient-reported form of the Multiple Sclerosis Neuropsychological Questionnaire (MSNQ) assesses perceived problems attributable to cognitive and neuropsychiatric symptoms. It is inconsistently related to objective cognitive performance in multiple sclerosis (MS), while strongly correlated with depression. We assessed whether the relationship between subjective and objective cognitive screening tools is moderated by disability. Furthermore, we investigated the MSNQ as a screening tool for both cognitive impairment and depression. 275 MS patients completed the patient-reported MSNQ, two‐question screening tool for depression and Symbol Digit Modalities Test (SDMT) and were divided into Expanded Disability Status Scale (EDSS) subgroups: Low 0.0–3.0, Medium 3.5–6.0, High 6.5–9.0. MSNQ scores correlated significantly with depression but not SDMT in all subgroups. After correcting for age, sex, education, EDSS and depression, MSNQ significantly predicted SDMT in the total group, but not the subgroups. MSNQ significantly predicted a positive depression and/or cognitive impairment screen in the total group and all subgroups. The relationship between subjective and objective cognitive screening tools is not influenced by physical disability. MSNQ scores are substantially influenced by depression, and reflect cognitive function to some degree. Patient-reported cognitive measures can be useful to identify patients requiring further (neuro)psychological assessment.

## Introduction

Multiple sclerosis (MS), the most common inflammatory and neurodegenerative disease in young adults, affecting more than two million people worldwide^1^, is characterized by substantial clinical heterogeneity. Yet, there is no valid multidimensional measure covering all aspects of disease, including the invisible burden of MS^2^. When time and resources are available, neuropsychological testing is included as a measure to capture the impact of MS. However, the relationship between the neuropsychological test results^3,4^ and the extent of cognitive difficulties experienced in daily life by the patient is not straightforward^4^.

Depending of the definition and the studied population, between 34 and 65% of persons with multiple sclerosis (PwMS) suffer from cognitive impairment. Cognitive processing speed and visual learning and memory are impaired in more than 50% of PwMS, while verbal and working memory impairment is seen in about 30%. Impairment of visuospatial abilities and executive functions are found less commonly, in about 20% of patients^5–8^. In view of the practical difficulties related to the use of extensive, neuropsychological test batteries^3,4^, a single tool, the Symbol Digit Modalities Test (SDMT) has been proposed. The SDMT is a simple and easily administered, objective cognitive screening test where the examinee reports numbers that match a symbol using a key at the top of the page. It is a sensitive, non-specific marker of cognitive function, with the potential to be a sentinel test for cognitive impairment in MS^9^. The test requires information processing speed in addition to language/verbal fluency, and nonverbal memory^10,11^. The SDMT is considered to be the best rapid assessment tool of cognition in clinical practice in MS^12^. While it may not reflect the general cognitive functioning of MS patients, recent data suggest a substantial correlation with the estimation of brain age based on brain volumetric parameters^13^.

The Multiple Sclerosis Neuropsychological Questionnaire (MSNQ) was developed as a screening test to assess perceived cognitive impairment and to a lesser extent personality and behavioural changes, without requiring professional expertise. Two forms of the MSNQ exist, namely the patient- and the informant-report (usually a family member) form. In this study we only consider the patient-report form. The test consists of 15 questions that are scored by frequency of symptoms^14^. The patient-report form weakly correlated with formal neuropsychological test results while the correlations of the informant-report form with formal neuropsychological test results were consistently more pronounced^14–17^. Increased MSNQ scores from the patient were attributed to depression^18^. These findings resulted in the proposal to consider only the informant-report form as a sensitive and validated screening tool for cognitive impairment^18^. Yet, recently reported associations of perceived cognitive difficulties with reduced employment and work performance^19,20^, health-related quality of life^19^, health-promoting behaviours^19,21^ as well as reduced thalamic, cortical grey matter^22^ and hippocampal volumes^23^, suggest a substantial impact of subjective cognitive complaints in PwMS, regardless of the link with depression. Furthermore, patient-reported cognitive performance measures have the important advantage of not needing trained personnel for their administration.

It is difficult to understand what contributes to the weak and variable associations between the patient-reported measures and the formal neuropsychological testing results. A potential explanation could be related to the variation in clinical disability as measured with the Expanded Disability Status Scale (EDSS^24^). While EDSS scores were not considered when selecting questions for the MSNQ^14^, later studies included patients with a wide variety of neurological disability^15–18,25^.

We hypothesise that the patient-reported MSNQ may perform better as a cognitive screening measure in patients with lower compared to higher EDSS scores. This is based on the assumption that several questions of the MSNQ, such as those pertaining to forgetting appointments, tasks and errands, may not apply to patients with higher EDSS scores. Increased physical disability is commonly associated with reduced activity and participation. Furthermore, these patients are at increased risk of failing formal cognitive tests^26,27^, even though they may not report cognitive symptoms^28,29^. Reduced awareness and lacking insight could explain why patient-reported MSNQ scores are not necessarily increased in a substantial proportion of patients with moderate to severe disability, despite objective cognitive impairment.

To evaluate our hypothesis, namely that the patient-reported MSNQ performs better as a cognitive screening tool in patients with lower compared to higher EDSS scores, we investigate correlations between MSNQ and SDMT scores in low, medium and high EDSS groups with and without correcting for other variables. As depression and cognitive impairment are highly prevalent, often overlapping and intricately linked in MS^12,30^, disentangling their respective contribution to the patient-report MSNQ scores is challenging. When aiming to assess the patient-reported MSNQ as a cognitive screening tool, potential confounding by depression needs to be taken into account. We hypothesize that the use of the patient-reported MSNQ as a screening tool for both depression and cognitive impairment may identify patients who need further (neuro)psychological assessment. We investigate this by assessing whether the MSNQ can predict a positive screen for depression and/or cognitive impairment.

## Materials and methods

### Participants

The study protocol, survey, patient information and informed consent were approved by the ethics committees at the University Hospital Brussels (B.U.N. 143201630261, 2016/357, 25 January 2017) and the National Multiple Sclerosis Center, Melsbroek (17/02, 24 January 2017) and all methods were performed in accordance with relevant guidelines and regulations. Informed consent was obtained from all participants. PwMS, aged 18 years or older, who were diagnosed with definite MS according to the McDonald criteria^29^ and registered in the EDMUS database from the University Hospital Brussels and the National Multiple Sclerosis Center, were included. Treatment status was not taken into account.

### Survey

1908 patients received a postal survey, including the patient-reported MSNQ and the two‐question screening tool for depression in MS^31^. The patient-reported MSNQ consists of 15 questions on perceived cognitive impairment and behavioural and personality changes on several aspects of daily life, with higher scores indicating a greater degree of perceived cognitive impairment^14^. The two-question screening tool for depression in MS consists of two questions, with a positive response on at least one of the questions indicating a positive screen for depression. It is a reliable tool for identifying depression in PwMS, with a sensitivity of 98.5% and a specificity of 87%. Moreover, a subthreshold depressive disorder was found in more than half of the patients with a false-positive score^32,33^. Since the scope of the study was to assess the value of the patient-reported MSNQ and the patients’ own assessment of cognition, the informant-reported MSNQ was not included in the survey. Patients were invited to contact a trained nurse by telephone in case of problems during completion of the questionnaires, to answer questions and administer the questionnaires together by telephone.

Sex, age, onset date (date of first symptoms), phenotype at onset, years of education, SDMT score within the last six months before or after the survey (administered by trained nursing personnel) and EDSS score (assessed by trained neurologists) were retrieved from the database. In the SDMT a page with a key pairing nine digits with nine symbols is presented, followed by several rows with only symbols. Patients have 90 s to orally report the correct digit for each symbol, based on the key. The score is based on the number of correct digits reported, with higher scores corresponding to a better information processing speed^10^. The EDSS is the most widely used clinical measure of disability in MS. The score ranges from 0 to 10 in half-point increments and is based on eight functional systems, which together reflect neurological impairment. This non-linear scale is heavily weighted towards physical disability and ambulation. Scores below 3 indicate a low impact of MS, while EDSS scores between 3 and 6 indicate moderate disability and EDSS scores above 6 correspond to severe disability^24^.

For a more detailed description of the methods used, we refer to previous publications on this data set^16,19^. Of note, both the SDMT and the two-question screening tool for depression in MS are used as screening tools in the context of this study, respectively for cognitive impairment and depression, since the goal of this study was to assess the value of the MSNQ as a screening tool rather than a measure of cognition and/or depression.

### Statistics

Patients were included if there was a full data set for the screening for depression, EDSS, MSNQ and SDMT scores. In case of an unanswered MSNQ question, this missing value was imputed by the mean item score. When more than one question of the MSNQ was not answered, this patient was excluded. The cohort was divided into three subcohorts according to EDSS scores: Low 0.0–3.0, Medium 3.5–6.0 and High 6.5–9.0. These cut-offs were based on the EDSS tertiles of our sample, as well as the severity of clinical and functional disease burden as estimated by experienced MS neurologists.

Results were reported as means with 95% confidence intervals (CI 95%), medians with ranges or as percentages. The relations between MSNQ, SDMT and depression were investigated through Pearson correlations in the total population and EDSS subgroups. Multiple linear regression analyses were performed to estimate the contribution of age, sex, education, depression, EDSS and MSNQ to the SDMT score in the total population and EDSS subgroups. Furthermore, we investigate the role of the MSNQ as a screening measure of both cognitive impairment and depression, by assessing its prediction of a positive screen for cognitive impairment and/or depression through a logistic regression analysis. A positive screen for cognitive impairment was defined as a normative SDMT-score (i.e. the raw SDMT is corrected based on the expected SDMT provided by a linear regression model trained on a group of healthy controls matched for age, years of education and sex^34^) of < − 1.5 standard deviations. A positive screen for depression was defined as answering ‘yes’ on at least one question of the two-question screening tool for depression in MS. A logistic regression analysis was performed to estimate the contribution of age, sex, years of education, EDSS and MSNQ to the prediction of a positive screen for depression and/or cognitive impairment. The performance of these models was assessed based on R^2^ and adjusted R^2^ and respectively t-tests and Wald tests were used to identify the significantly contributing explanatory variables. Of note, EDSS was not added as an independent variable in the EDSS subgroup models. Finally, group differences in MSNQ scores between cognitively impaired versus cognitively preserved, depressed versus non-depressed and impaired versus non-impaired (i.e. a positive screen for cognitive impairment, depression or both) patients were assessed visually through boxplots. A two-sided test with a type I error probability of 0.05 was used for all analyses. The Bonferroni method was used to correct for multiple comparisons^35^.

## Results

### Population characteristics

277 patients met the inclusion criteria of our study. After visual inspection two patients with SDMT values of above 80 were removed from the data set, resulting in a total of 275 included patients. These SDMT outliers were patients who underwent monthly SDMT evaluations in the context of their treatment with natalizumab, which has led to an important learning effect due to repeated test exposure. In Table 1 the characteristics of the three EDSS subgroups and the total population are listed. The mean SDMT score was significantly different between the EDSS subgroups, with the low EDSS group scoring the highest and the high EDSS group the lowest. There was no significant difference in the mean MSNQ score among the three groups.

**Table 1:** Population characteristics.

Baseline characteristics	All	Low EDSS	Medium EDSS	High EDSS
	275	80/275	125/275	70/275
**Age**				
Mean [CI 95%]	52.5 [51.4–53.6]	46.4 [44.5–48.4]	53.6 [52.1–55.0]	57.4 [55.1–59.7]
Median [range]	53.0 [23.0–78.0]	49.0 [23.0–66.0]	54.0 [27.0–77.0]	59.0 [31.0–78.0]
**Sex**				
Female, n/N (%)	176/275 (64.0%)	56/80 (70.0%)	83/125 (66.4%)	37/70 (52.9%)
**Onset type**				
Relapsing, n/N (%)	207/275 (75.3%)	70/80 (87.5%)	94/125 (75.2%)	43/70 (61.4%)
**Disease duration**				
Mean [CI 95%]	18.9 [17.9–19.8]	14.4 [12.9–15.9]	19.2 [17.8–20.5]	23.4 [21.3–25.6]
Median [range]	18.0 [2.0–49.0]	14.0 [2.0–33.0]	18.0 [2.0–45.0]	23.0 [4.0–49.0]
**Years of education**				
Less than 12 years, n/N (%)	25/275 (9.1%)	7/80 (8.8%)	9/125 (7.2%)	9/70 (12.9%)
Between 12 and 15 years, n/N (%)	124/275 (45.1%)	32/80 (40.0%)	63/125 (50.4%)	29/70 (41.4%)
More than 15 years, n/N (%)	126/275 (45.8%)	41/80 (51.2%)	53/125 (42.4%)	32/70 (45.7%)
**Depressed**				
Depressed, n/N (%)	123/275 (44.7%)	36/80 (45.0%)	57/125 (45.6%)	30/70 (42.9%)
**EDSS**				
Mean [CI 95%]	4.6 [4.4–4.9]	2.0 [1.9–2.2]	4.9 [4.7–5.0]	7.3 [7.1–7.4]
Median [range]	4.5 [0.0–9.0]	2.0 [0.0–3.0]	5.0 [3.5–6.0]	7.0 [6.5–9.0]
**MSNQ**				
Mean [CI 95%]	21.9 [20.7–23.0]	22.2 [20.0– 24.5]	22.4 [20.7–24.0]	20.5 [18.2–22.9]
Median [range]	22.0 [0.0–53.0]	22.0 [0.0–53.0]	23.0 [1.0–49.0]	17.0 [0.0–47.0]
**SDMT**				
Mean [CI 95%]	47.0 [45.7–48.3]	55.3 [53.4–57.2]	46.8 [45.1–48.5]	38.0 [35.5–40.5]
Median [range]	48.0 [15.0–74.0]	55.5 [23.0–74.0]	48.0 [15.0–72.0]	39.0 [15.0–67.0]
**Positive screen for depression and/or cognitive impairment**				
Impaired, n/N (%)	153/275 (55.6%)	42/80 (52.5%)	67/125 (53.6%)	44/70 (62.9%)

*CI 95%* 95% confidence interval, *EDSS* Expanded Disability Status Scale, *MSNQ* Multiple Sclerosis Neuropsychological Questionnaire, *SDMT* Symbol Digit Modalities Test.

**Figure 1:**
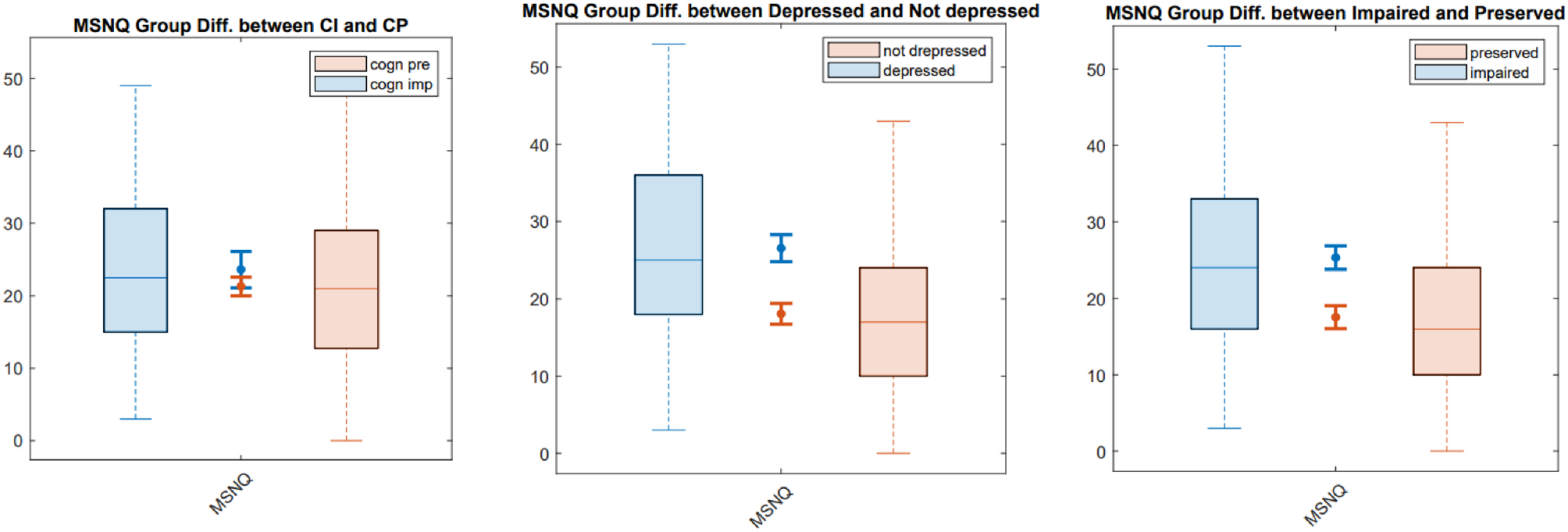
Group differences in MSNQ scores in cognitively impaired and depressed patients. Box plots of the MSNQ scores of cognitively impaired versus cognitively preserved patients (first panel), depressed versus non-depressed patients (second panel) and impaired (i.e. cognitive impairment and/or depression) versus non-impaired (i.e. no cognitive impairment or depression) patients (third panel). *Diff.* differences, *MSNQ* Multiple Sclerosis Neuropsychological Questionnaire, *CI* cognitively impaired, *CP* cognitively preserved, *impaired* depression and/or cognitive impairment, *preserved* no depression or cognitive impairment.

**Figure 2:**
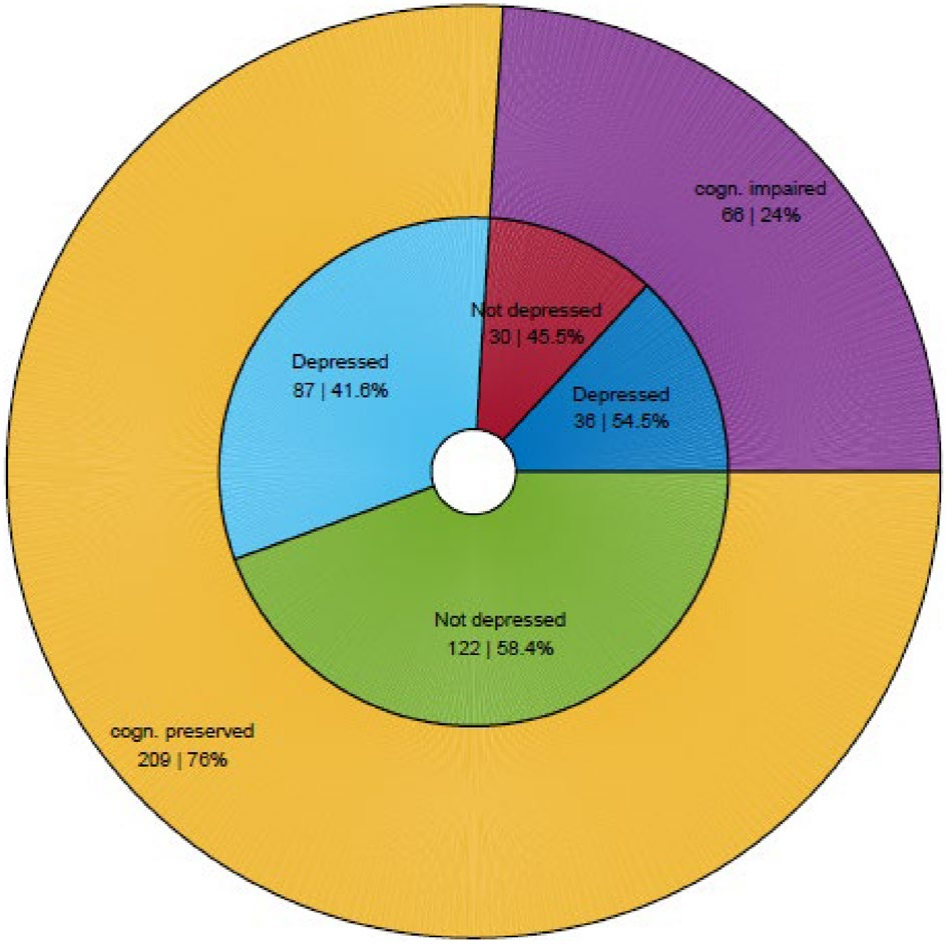
Presence of a positive screen for depression and cognitive impairment in the total population. The outer circle shows the proportion of cognitively impaired versus preserved patients, while the inner circle shows the proportion of depressed versus non-depressed patients in those two groups. *Cogn.* cognitively.

Mean MSNQ scores were significantly different in depressed versus non-depressed and impaired versus non-impaired patients (i.e. a positive screen for cognitive impairment, depression or both), but not in cognitively-impaired versus cognitively-preserved patients in the total population (see Fig. 1) and all EDSS subgroups (not shown). The proportion of patients in the total population that is cognitively impaired versus preserved and depressed versus non-depressed is depicted in Fig. 2.

### Correlations

As listed in Table 2, there were no significant correlations between MSNQ and SDMT scores in the total population or any of the EDSS subgroups. MSNQ scores significantly correlated with depression in the total population and all EDSS subgroups.

**Table 2:** Pearson correlations in the different EDSS subgroups.

		All		Low EDSS		Medium EDSS		High EDSS	
Var1	Var2	*r*	*p*-value	*r*	*p*-value	*r*	*p*-value	*r*	*p*-value
MSNQ	SDMT	− 0.070	0.244	− 0.136	0.229	− 0.116	0.199	− 0.083	0.496
MSNQ	Depression	0.365	< 0.001*	0.372	0.001*	0.371	< 0.001*	0.345	0.003*

Correlations that are significant (with Bonferroni correction, *p* < 0.05/8) are marked with *.

*EDSS* Expanded Disability Status Scale, *MSNQ* Multiple Sclerosis Neuropsychological Questionnaire, *SDMT* Symbol Digit Modalities Test.

### Regression analyses

To estimate the combined contribution of age, sex, years of education, EDSS, depression and MSNQ to the SDMT score in the total population and the EDSS subgroups, multiple linear regression analyses were performed. Age, EDSS and MSNQ significantly and negatively contributed to the predicted value of the SDMT model of the total population (see Table 3). An increase of 1 year in age corresponded to a decrease of 0.20 points, an increase of 1 EDSS-point corresponded to a decrease of 2.93 points and an increase of 1 MSNQ-point corresponded to a decrease of 0.13 points in the predicted SDMT score respectively. None of the other variables significantly contributed. The contribution of MSNQ scores was no longer significant in the EDSS subgroup analyses. Of note, EDSS was not added as an independent variable in the EDSS subgroup models.

**Table 3:** Multiple linear regression analysis of SDMT in the total population.

N	Test statistic	R^2^	R^2^_adjusted_	r
275	26.69	0.33	0.32	0.580

Variables		b (CI 95%)	b_0_ (CI 95%)	*p*-value
Age	− 3.10	− 0.20 ± 0.11	− 0.17 ± 0.09	0.002*
Sex: male	− 0.11	− 0.15 ± 2.25	− 0.01 ± 0.17	0.913
Years of education	0.30	0.06 ± 0.31	0.02 ± 0.08	0.748
EDSS	− 8.86	− 2.92 ± 0.54	− 0.48 ± 0.09	< 0.001*
Depression	15.90	− 0.68 ± 2.32	− 0.05 ± 0.18	0.627
MSNQ	− 2.33	− 0.12 ± 0.10	− 0.11 ± 0.09	< 0.050*

Statistically significant predictors (p < 0.05) are marked with *.

*CI 95%* 95% confidence interval, *EDSS* Expanded Disability Status Scale, *MSNQ* Multiple Sclerosis Neuropsychological Questionnaire.

To estimate the contribution of age, sex, years of education, EDSS and MSNQ to the prediction of a positive screen for depression and/or cognitive impairment, a logistic regression analysis was performed. EDSS, years of education and MSNQ significantly contributed to the prediction of a positive screen for depression and/or cognitive impairment of the total population (see Table 4). Furthermore, years of education was a positive predictor in the medium and high EDSS subgroups and MSNQ was a positive predictor in all subgroups. Of note, EDSS was not added as an independent variable in the EDSS subgroup models.

**Table 4:** Logistic regression analysis of the prediction of a positive screen for depression and/or cognitive impairment in the total population.

N		R^2^	R^2^_adjusted_	
275		0.15	0.13	

Variables	Test statistic	b (CI 95%)	b_0_ (CI 95%)	*p*-value
Age	0.005	− 0.00 ± 0.02	− 0.01 ± 0.24	0.943
Sex: male	0.89	0.27 ± 0.47	0.27 ± 0.47	0.347
Years of education:	11.05	0.14 ± 0.07	0.47 ± 0.23	0.001*
EDSS	8.67	0.21 ± 0.12	0.45 ± 0.25	0.003*
MSNQ	33.16	0.08 ± 0.02	0.93 ± 0.27	< 0.001*

Statistically significant predictors (p < 0.05) are marked with *.

*CI 95%* 95% confidence interval, *EDSS* Expanded Disability Status Scale, *MSNQ* Multiple Sclerosis Neuropsychological Questionnaire.

## Discussion

We did not find significant correlations between MSNQ and SDMT scores in the total population or any of the EDSS subgroups. So, our hypothesis that the patient-reported MSNQ performs better as a cognitive screening measure in patients with low compared to medium and high EDSS scores could not be confirmed. However, MSNQ scores contributed significantly and negatively to the prediction of SDMT scores after correcting for age, sex, education, EDSS and depression. This was the case for the total population only, not for the EDSS subgroup analysis (possibly due to smaller sample size in the subgroups). These findings suggest that patient-reported MSNQ scores reflect cognitive functioning, as measured by the SDMT, at least to some degree. Furthermore, MSNQ scores contributed significantly to the prediction of a positive screen for cognitive impairment and/or depression after correcting for age, sex, years of education and EDSS, both in the total population and all EDSS subgroups.

Our findings confirm the association of patient-reported MSNQ scores with depression across all EDSS subgroups. Furthermore, while MSNQ scores contributed significantly to the prediction of a positive screen for cognitive impairment and/or depression, this contribution was mainly driven by the association with depression. More specifically, mean MSNQ scores were significantly different in depressed versus non-depressed and impaired versus non-impaired patients (i.e. a positive screen for cognitive impairment, depression or both), but not in cognitively-impaired versus cognitively-preserved patients. The association of the patient-reported MSNQ scores with depression is usually considered to be an important limitation when using it as a measure of cognition^14–18^. However, accurately measuring the cognitive issues caused by MS is challenging^36^ and the interplay between cognitive performance and depressive symptoms is complex. Cognitive impairment and depression are highly prevalent and underdiagnosed in PwMS^12,30^ and both have been associated with increased disability progression^37,38^. Depression also influences information processing speed, memory and executive function in PwMS and affects health behaviours and leisure activities, thereby reducing potential protective effects on cognition^39,40^. Previous studies with the Perceived Deficits Questionnaire have found evidence that subjective cognitive impairment reflects subtle declines in cognitive processing speed and memory independent of mood^29^ and that correlations between patient-reported memory and hippocampal volumes were maintained after controlling for depression^23^. While the patient-reported MSNQ in our study was clearly influenced by depression, it also reflects a marker of cognitive function, as indicated by our multiple linear regression model of SDMT. These findings are in line with previously described results^25^. Therefore, reduced subjective cognitive performance and depressive symptoms should both be taken seriously and urge further investigation, especially when considering the important impact of these symptoms on daily life^19–21,30,38–40^. The patient-reported MSNQ, while not a reliable screening tool for cognitive impairment alone, can be useful in identifying patients who are at risk of being depressed and/or cognitively impaired, and who thus require further (neuro)psychological assessment.

The patient-report MSNQ has important shortcomings as a screening measure for cognition, as evidenced by its limited predictive value in our regression models. Since more reliable tools to screen for depression and cognitive impairment separately do exist, we do not propose the MSNQ as an alternative for these tests. Instead, we want to emphasize that a patient-reported cognitive performance measure can be a useful screening instrument to identify patients who may not feel confident in their cognitive capacities, be it due to cognitive impairment or depression or a combination of both. We argue that the association of patient-reported screening measures with depression does not invalidate the usefulness of these measures. Notably, depression and cognitive impairment in MS are inherently linked and underdiagnosed^12,30^. Furthermore, the main advantage of the MSNQ is its ease of administration. Since trained personnel is not needed for the administration the patient can fill in this single short questionnaire at home or in the waiting room before the consultation. Finally, when used in combination with the informant-report form of the MSNQ, the patient’s ability to estimate his own level of impairment can be assessed^28^. An important next step in this research field would be the development of a new patient-reported screening tool for MS that can identify cognitive impairment more reliably, while taking depression into account.

Important strengths of our study are that, as far as we know, we are the first to assess the relationship between measures of objective and subjective cognitive performance in different EDSS groups. Furthermore, participants were recruited from two centres, representing a broad range of EDSS scores. A limitation is the cross-sectional study design. Moreover, as already discussed^16,19^, there was a moderate response rate to the survey, with a possibility of selection bias. Only 24% of our patients had a positive screen for cognitive impairment, which is lower than the expected 34 to 65%^5–8^. This could be due to the fact that patients were recruited either in an academic hospital (University Hospital Brussels) or a reference institute (National MS Center Melsbroek), where they regularly undergo cognitive testing, possibly resulting in a learning effect. The proportion of patients in our study that had a positive screen for depression on the other hand corresponded to the expected prevalence of around 40%^30^. Our patients were also older and had a longer disease duration and higher EDSS scores compared to other studies on the MSNQ^14,15,17,18,25,28^, and had a higher education level compared to a large sample study of Belgian patients with MS^41^. Another weakness is that the MSNQ was sent by post and performed by the patient at home, which makes completion of the survey in a controlled environment impossible. Furthermore, the SDMT was assessed within six months before or after the survey, which could introduce bias since SDMT and MSNQ scores were not obtained at the same time. Finally, another limitation is that only one objective cognitive test was carried out. For the assessment of the value of the MSNQ as a screening tool for cognitive impairment, we used the SDMT. The SDMT is considered to be the golden standard to screen for cognitive impairment in MS^12^. However, it mainly assesses information processing speed^10^, and could therefore fail to detect impairment in other domains, such as verbal memory and executive functions.

In summary, we found that correlations between MSNQ and SDMT scores are not influenced by the patient’s level of physical disability. We were able to confirm patient-reported MSNQ scores are substantially influenced by depression, but nonetheless reflect cognitive impairment to some degree. Since both depression and cognitive impairment are underdiagnosed in PwMS and given the important impact of subjective cognitive symptoms on daily life, patient-reported cognitive measures such as the patient-reported MSNQ can be useful screening tools for the identification of patients requiring further (neuro)psychological assessment and interventions.

## Data Availability

Data is available upon reasonable request to the senior authors.
